# Physical activity in light of affordances in outdoor environments: qualitative observation studies of 3–5 years olds in kindergarten

**DOI:** 10.1186/s40064-016-2565-y

**Published:** 2016-06-30

**Authors:** Kathrine Bjørgen

**Affiliations:** Queen Maud University College of Early Childhood Education, Thrond Nergaardsveg 7, 7044 Trondheim, Norway

**Keywords:** Kindergarten children, Affordances, Physical and social environment, Physical activity

## Abstract

This article examines the characteristic of affordances of different outdoor environments, related to the influences of children’s physical activity levels. Qualitative observation studies in a Norwegian kindergarten were conducted of 3- to 5-year-olds into the natural environment and in the kindergarten’s outdoor area. An ecological approach was important from both an analytical and theoretical point of view, using concepts from Gibson’s (The ecological approach to visual perception. Houghton Mifflin Company, Bosten, 1979) theory of affordances. The concepts of affordances in an environment can explain children’s movement behaviour. The findings reveal that situations with high physical activity levels among the children are more often created in natural environments than in the kindergarten’s outdoor environment. Natural environments offer potential qualities that are a catalyst for physical activity. The study shows that certain characteristic of the physical outdoor environment are important for children’s opportunities and inspiration for physical active play. The findings also show that social possibilities and opportunities, human interactions, in the environment have the greatest influence on the duration and intensity of physically active play. The need for knowledge on physical and social opportunities in outdoor environments, educational practice and the content of outdoor time in kindergartens should be given greater attention.

## Background

We live in a time when there is growing concern about low physical activity levels and health-related problems in young children. A sedentary lifestyle is a global problem (Directorate 2014; Gray et al. 2015; WHO 2010). Research shows that pre-schoolers’ physical activity level is characterized as sedentary throughout their preschool day (Brown et al. 2009; Dowda et al. 2004; Gubbels et al. 2011; Tucker 2008). It is well documented that physical activity enhances good health, provides energy and is an important means in the prevention of various forms of lifestyle illness (Directorate 2014). The Norwegian directorate of Health (Directorate 2014) recommendations provide that children and young people should be physically active at least 1 h a day, and with moderate to high intensity. Playing outdoors, particularly in natural environments, increases children’s physical activity levels, while also having several positive effects on children’s health, wellness, learning and development (Gray et al. 2015; Herrington and Brussoni 2015; Spencer and Wright 2014). Landscape features and outdoors environments in the kindergarten are seen as an arena for promoting children’s physical activity, -motor development and risky play (Fjørtoft 2000; Giske et al. 2010; Sandseter 2009). The natural environment is a valuable source for diverse learning and diverse play habitats for children (Fjørtoft 2004). Natural environments represent spaces where children see opportunities and challenges according to the qualities of the natural setting, and then use them functionally (Fjørtoft 2000). In an interview study of kindergarten practitioner’s, they attached importance to how trips in nature create enthusiasm and motivation for children’s physical play (Bjørgen and Svendsen 2015). According Spencer and Wright (2014) children display highest level of physical activity on playgrounds that have a combination of manufactured equipment and natural materials. Supporting diverse affordances for play through a well thought out design of play spaces can encourage children’s engagement in play, where physical activity as a positive side effect (Herrington and Brussoni 2015).

Most children in kindergarten in Norway spend a relatively large amount of time outdoors and have access to natural environments and large outdoor play spaces (Moser and Martinsen 2010; Stokke 2011). The Framework Plan for the content and task of kindergartens (Ministry 2011) underlines the importance of varied physical activity, both indoors and outdoors. Research in Norwegian kindergartens can refer to different results in the physical activity level of children (Giske et al. 2010; Stokke et al. 2014; Storli and Hagen 2010). However, it is fairly safe to assume that child’s physical activity levels will depend on which institution they attend environmental and individual resources. Interpretations and comparisons of findings on this issue are challenging (Stokke et al. 2014). Describing the context is crucial to understand children’s enjoyment and involvement in physically active play. The definition of physical activity in kindergarten children, should be seen in a context of play, defined as at a *moderate or high level of physical activity in a context of playing with energy well above the rest level* (Osnes et al. 2010). A context of play means that the play in itself is the goal, that it is voluntary and driven by the child’s internal motivation. Traditionally, the time spent outside in Norwegian kindergartens has been considered as constituting an arena for free play. Kallestad and Ødegaard (2014) show that the activities in kindergartens are typically not planned and are the result of the children’s own initiatives (80 % non-planned activities). This may be because the concept of free play is well embedded in Norwegian kindergartens culture and tradition.

The quality of the physical and social environment that occurs outside may both be considered to have supportive or impeding impact on children’s activity levels. Children’s day-to-day lives in kindergarten may be understood in a socio-cultural context, where social interplay and interaction in relation to the environment are important elements in the children’s development (Rogoff 2003; Vygotsky 1978). The quality of social interplay, friends, responsive adults and safe environments may have an impact on involvement in activities. According to Rogoff (2003), children should be guided in a sensitive manner through guided participation, where the children and the adults should have a mutual relationship. Imitation and joint attention may also be related to, influence and explain human involvement in an activity (Tomasello et al. 2005; Tomasello and Haberl 2003). Earlier findings from the same data material used in this article showed that the well-being and involvement of 3- to 5-year-olds in physical play are created through social relationships, freedom to act and the challenges and opportunities for variation in the outdoors environment (Bjørgen 2015).

## Analytical and theoretical approach

The choice of theoretical and analytical approach was made according to the empirical data material. An ecological perspective may explain how the physical environment impacts children’s movement behaviour (Gubbels et al. 2011). Gibson’s (1979) theory of affordances links together the perceptual and motor systems, and explains how the physical environment is interpreted by individuals as invitations to behavior and possibilities for action. Gibson’s theory has been considered as an directly perceived model of ecological perceptions and its focus on physical affordances in the environment. Heft (1988) further developed the theory of affordances as a means to describe how children perceive functionally significant properties in their environment and adapt their actions according to their own resources such as their strength, competence or fear. Kyttä (2006) connect a close relationships between children’s degree of independent mobility licence and actualization of affordances in the environments. Kyttä (2004) refers to *potential affordances*, which are opportunities inherent in the qualities of the environment (possible affordances of an environment or object), and *realised affordances*, which are opportunities the children use in the environment. The extent of potential affordances is defined by the individual’s qualities, such as physical skills and bodily proportions, as well as social needs and personals intention (Kyttä 2003). Therefor, the potential affordances can be different for each individual, group, people and situations. Environments involving a positive interrelationships between mobility licence and actualizing affordances also functions as a zone of proximal development (Vygotsky), which means that children are presented with a series of graduate zones of challenges slightly above their current levels of functioning (Kyttä 2006). Few mobility licences make it impossible to actualize affordances.

Herrington et al. (2007) describe seven criteria for what characterises outdoor play spaces that support the development of young children and integrate the unique qualities of playing outdoors, called the Seven Cs (7Cs). The criteria describe outdoor play spaces that describe the play affordances (Herrington and Brussoni 2015; Herrington et al. 2007). The 7Cs relate *character* to the characteristics of a physical environment, properties and general feelings and impressions (light, sound, colours or soft/hard materials), which are found in the environment and which influence people. The *context* involves how the play space has physical and visual interaction with its surroundings, in the play space and in relation to other surrounding areas. *Connectivity* refers to connections (physical and visual) in the play space that may create understanding of time, space and movement opportunities. This may be the link between outdoor and indoor space. *Change* relates to how the play space is perceived and used through changes in seasons, over the course of the year and in the age groups. *Chance* refers to the opportunities children have to create, manipulate, shape and leave imprints using different materials (sand, water, clay, snow, ice). Chance in the play space creates spontaneous challenge, manipulation and discovery, allowing the play space to be used in different ways according to the child’s size, age and resources. The *clarity* of the environment relates to readability and spatial legibility, and to children’s perceptual ideas of how the play space can be used. The environment and materials (hard/soft surfaces, noise in the neighbourhood, placement of toys and play apparatuses) influence how children play. The term *challenge refers* to how the play space creates physical and cognitive challenges and risk-taking. While the 7Cs were developed in Canada, the criteria are general so in this study they can be applied to understand how children use their outdoor play spaces in a Norwegian context to create physical activity.

Social affordances are a subcategory of affordances, namely possibilities for social interaction offered by the environment (Rietveld et al. 2013). Clark and Uzzell (2006) utilize the term environmental affordances to have an equally strong social dimension; the affordances may be direct (from physical environment) or indirect (mediated by the social presence or behaviour of others). Charles (2012) argues that social affordance refers to opportunities in the environment that promote social relationships and interaction. Social interaction refers to an individual’s opportunities in the environment, to responses, social accessibility and the ability to adapt to social changes in the environment (Charles 2012). Clark and Uzzell (2006) state that to have the ability to interact with the environment, individuals have to be able to perceive its social meaning. Rietveld et al. (2013) claim that we are selectively responsive to one affordance rather than another; we are motivated by the situation. Individuals perceive the environment holistically and do not perceive or utilize social and physical aspects of the environment separately and in isolation from each other (Clark and Uzzell 2006).

In this article, the “affordances” concept is related to how children in kindergarten use physical and social invitations and opportunities in different outdoor environments, considered together with children’s physical activity levels.

## Research question

The aim of this study is to examine how the affordances in two different outdoors environments explain children’s level of physical activity. The research question is: *What is the relation between environmental affordances and physical activity level among 3–5* *year olds? In light of two different types of kindergarten outdoor environments; (1) trips in a natural environment and (2) the kindergarten’s outdoor play space.*

## Methods

A number of measuring methods and instruments have been used in research on children and physical activity (actigraph/accelerometer, heart rate, questionnaires, observation). Stokke et al. (2014) have compared different measuring instruments and suggest that *seeing* the child through observation may give good indications of a child’s physical activity level. The methodology of observations and video recordings represented a good approach for this study, where the aim is to analyse, interpret and describe affordances in different physical outdoor play spaces and their importance for children’s physical activity levels.

### Participants and design

The data material was collected at a kindergarten in central Norway. Initially the kindergarten director was contacted by telephone, and then a letter providing information about the project was sent to the director. The kindergarten indicated it was interested and willing to participate. The selection criterion employed by the researcher was that the children have experience with outdoors activities in their kindergarten. The kindergarten is an ordinary/traditional centre (not a nature/outdoor kindergarten), which fallow the purpose, values and task stated in the day care program of Framework Plan of Norwegian Kindergarten (Ministry 2011). The choice of this criterion was based on the rationale that the observations could reflect routines, outdoor time, framework and content which could be approximately similar to many Norwegian kindergartens. The focus areas of the kindergarten are safety, learning, teaching methods and outdoor activities. The employees consist of educated preschool teachers and assistants. The observations data were conducted in the 3–5 years department, 24 children, 14 girls and ten boys. The children played in the *kindergarten’s outdoor play space* every day for about 3 h, and each week groups would go on trips (one or two times) *to various natural environments*. On trips in the nature the children were often divided into smaller groups related to ages, in the outdoor play space they were all together. The number of children who participated in the observation varied, due to sickness absenteeism and which group observed.

### The context of the kindergarten

The kindergarten is situated in central Norway in a suburb of Trondheim, and in close proximity to forests, hiking areas, paths, skiing paths and open fields, all of which are used. The kindergarten’s outdoor area measures 3681.3 m^2^ (specifications obtained from Trondheim local authority), and is playground with complexity and variety of elements, furnished with *manufactured equipment*; outdoor toys (buckets, shovels, trucks, balls), swings, sandboxes, climbing racks, and *natural materials*; small trees, a varied surface of grass, sand and asphalt, and varied terrain with small hills. The destination for excursions in diverse natural landscape environment is approximately 300–700 m from the centre. The landscape has different qualities to meet children’s need for varied play and physical-motor challenges. One type of natural environment was open fields suitable for tobogganing, running and playing on skis. Another natural environment consisted of woods with various possibilities for playing. Trips were made to the natural environments all year round.

### Observations

Observations were made with video recording through the different seasons of the year for 20 days, 10 days on trips in a natural environment and 10 days in the centres play space. A total of 50 h of direct observation (30 h without a JVC video camera and 20 h with a video camera) was conducted. The observations were naturalistic, non-participatory inspections of the children’s play in their natural outdoors kindergarten (and hiking vicinity) setting. The researcher first visited the children in the kindergarten to familiarize themselves with the setting and the people at the kindergarten. This technique enabled the researcher to be open, discovery oriented and inductive (Patton 2002). Gradually, the observations become more structured and concrete, concentrating on the essential physically active play situations. In the end, a saturation point was reached in which the observations did not provide any additional examples of the children’s physically active play.

This article presents findings from 6 days randomly selected, three from a natural environment and three from the play space in the kindergarten. This was done to reduce the amount of data and to make it more easily manageable. The context and the process of each observation were described accurately. Field notes were used, where the date, time, place, actors, activity, objects, acts, events, purpose and feelings were described. The video observations were transcribed by the researcher.

### Ethical considerations

There are special ethical issues to consider when researching kindergartens. For example, informed consent must be obtained from the research subjects, and in this study informed consent was obtained from the parents. A detailed description of the research project, its aim, methodology and implications for the kindergarten was sent to the directors, the preschool staff and the parents. The parents gave their written consented stating that their child could be observed and that video-recordings could be made. Another ethical issue is the question of confidentiality and anonymity. The description of the research project included a guarantee of full anonymity during data collection and publication. Participation was voluntary and the project was approved by Norway Social Science Data Services (NSD). All the data material, which was been treated confidentially and anonymously, will be deleted when the project is concluded.

Observation as a method requires critical reflection. Truth may be construed and institutionalised according to whom the observer is, and according to the experiences, values and self-understanding of the observer (Stokke et al. 2014). Løkken (2012) suggests that a researcher’s body is in interaction with the surrounding world and part of the observation context, thus exerting influence on what is observed. To help in reflecting on one’s self-understanding when assessing the physical activity levels of children, two assistants were used (one kindergarten staff member and a project mentor) to code the children’s levels of physical activity. The assistants assessed the children’s physical activity levels independently. Finally, coding of the children’s levels were compared and regulated according to a common understanding.

### Observation manual

Coding of the physical activity levels of children was assessed and adapted using the *Observational System for Recording Physical Activity in Children*—*Preschool Version* (OSRAC-P) manual, developed by Brown et al. (2012). OSRAC-P includes observable categories of coding to indicate children’s physical activity level/intensity and type. The physical activity level categories are in five levels: 1 = stationary/motionless, 2 = stationary with limb or trunk movement, 3 = slow, easy activity, 4 = moderate movement, 5 = fast movement.

The use of OSRAC-P was modified to identify the level of children’s physical activity in situations of physically active play, researcher did not use a statistical procedure. Each child who participated in the physical-play situation was assigned a level (1–5) of physical activity, assessed every 15 s within approximately 2 min, depending on the duration of the play. The highest indicated level of physical activity was registered for each participating child, thereafter an average level of physical activity was identified in the situations. The researcher endeavoured to render a holistic picture of the specific situation with physical play. Graded coding was used, and if a child’s activity level varied between 3 and 4, it was assigned the 3.5 level.

### Analysis of the data

The field record and transcriptions of the observations from the video recordings constituted the data material which formed the basis for the analysis. Thematic analysis (Boyatzis 1998; Braun and Clarke 2006) was used in the process by coding, examining and recording patterns (themes, characters) within data. Thematic analysis is performed through the process of coding in six phases; familiarization with data, generating initial codes, searching for themes among codes, reviewing themes, defining and naming themes, and a final report/discussion (Braun and Clarke 2006). The first phases of coding were assessing and identifying the children’s level of physical activity in different play situations. Figures were used as an analytical tool helped to discern patterns, differences and similarities in the data material, which laid foundations for the qualitative analysis of the affordances. Thereafter the researcher searches to identify characteristics (themes) of affordances within the data. The theory of affordances and criteria from the 7Sc (Herrington et al. 2007) were used in the analysis process.

## Findings

**Fig. 1:**
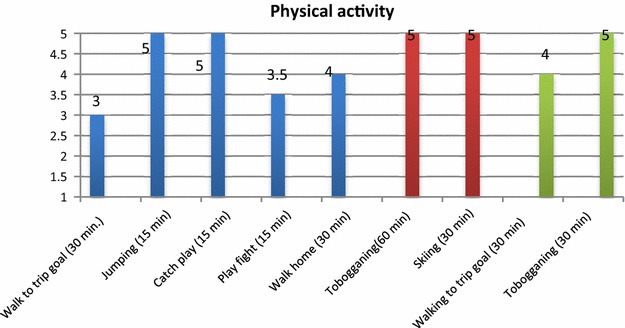
Illustration of the content, time and level of children’s physical activity levels from situations with physical play, on 3 days (*different colours*) in trips in natural environments. Average level of physical activity: 4.4

**Fig. 2:**
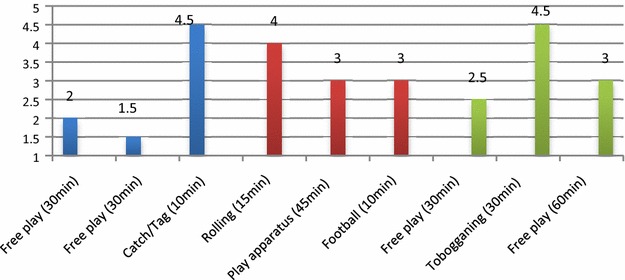
Illustration of the content, time and level of children’s physical activity levels from situations with physical play, on 3 days (*different colours*) in the day-care centre’s outdoor play space. Average level of physical activity: 3.1

Physical active play situations from six randomly selected days are presented; three from a natural environment (Fig. 1) and three from the play space in the kindergarten (Fig. 2). The participants were assigned a physical activity level, and these situations were then merged into an average level for each situation as shown in Figs. 1 and 2. The three different days were systematized into different colours (blue, red, green), and shows the content, time and level of physical play, as shown in Figs. 1 and 2.

The findings from 3 days with trips in a natural environment show that the children had an average level of more than an hour with moderate to high physical activity (4.4) each day. This satisfies the Directorate of Health’s recommendations.

In the same way as for trips in the natural environment, the content, time and level of situations with physical play in the outdoor kindergarten play space over 3 days are analysed in Fig. 2.

The findings from 3 days in the centre’s outdoor space showed on average low to medium physical activity at level 3.1. Some situations (catch/tag, rolling, tobogganing) of short duration showed a high physical activity level (4.5).

The findings on average showed a higher physical activity level (level 4.4) in situations with physical play during 3 days on trips in a natural environment compared to 3 days in kindergarten’s outdoor space (level 3.1). In free play in the outdoor space the observations showed lower physical activity levels (levels 1–3) than during parts of the play in the outdoor environment where the children played together (catch/tag, rolling, tobogganing, levels 4–5). *Free play* is when children play alone or with other children without adults initiating, facilitating or organising the play. *Playing together* is when several children play together where adults are present and the play has shared content. Playing together is voluntary, initiated by children or adults, and the children determine how much they wish to participate and how they move.

### Qualitative analysis of description of a day in natural environment and a day in of the play space in the kindergarten

To answer the research question a qualitative description and analyse of the physical play situations were conducted to interpret *the relation between environmental affordances and physical activity level among 3–5* *year olds, in light of two different types of kindergarten outdoor environments; (1) trips in a natural environment and (2) the kindergarten’s outdoor play space.*

#### A day in a natural environment

Walking to and returning from a trip destination in a natural environment created spontaneous events based on the character and context (7Cs) of the nature. The children displayed interest and enthusiasm, and became involved in natural materials on the ground. The chance (7Cs) of elements of snow, ice, cones, water, twigs, sticks and living organisms (plants, small animals, insects) invited involvement, movement, social interaction and shared experiences. Connectivity (7Cs), physical and visual connections in the nature create movement opportunities, physical activity and social opportunities and interactions. Two situations will be described about walking to a trip destination in nature and back (ten 4- and 5-year-olds, two adults, autumn weather).

Situation 1, Walking to a destination in the forest, 30 min; “*The children are walking at different speeds, they walk, stop, run a bit, inspect/interact with elements of nature (water ice, twigs). The children walk on a path in a line in the forest. An adult is at the head of the line, the other adults at the back. The staff determine the speed, and are focused on reaching the destination for the trip*” *(*transcriptions). The physical activity was assessed at levels to 3.

Situation 2, on the *return trip* another path is chosen in the terrain (no path); the children are involved in determining direction, a type of orienteering game. They alternate in leading the line, and they are allowed to help decide which direction to take. The staff ask the children questions: such as “*Which way do you think the kindergarten is? Where do we go?”* The children point and make suggestions*. “Can you, X, show the way?”* The physical activity was assessed at levels 3 to 4.

##### Interpretation 

Walking to and returning from a trip destination is physically demanding on physical skills for many children. Situations 1 and 2 shows that different staff involvement influence the children’s mobility licence to realise affordances. In situation 2 spaces was created for being involved, and to help decide where and how to move through varied terrain. The staff’s tolerance and regulation of the children’s independent mobility license and co-determination influence the children’s risk taking and physical activity level. This provided them with physical motor challenges they otherwise would not have experienced. Situation 1; walking on a path in line reduced the amount of spontaneous movement, and the physical activity level was assessed to medium. The character, context and connectivity (7Cs) of nature (the terrain, surface, loose and malleable objects) challenge the movement repertoire of the children and were a catalyst for physical activity. The staffs social afford to involve the children in decision making where to move, create motivated actions for movement.

In the trip, at the destination for the walk, the preschool teacher and the children discover a small knoll in the terrain and start jumping from it.

Situation 3, Jumping from a small knoll (15 min); “*high physical*-*motor levels are created, the children jump down and run back up. They talk, shout and laugh. Three of the girls jump together and try to land in differing ways. They hold hands and try to jump together from the small knoll. There is laughter. They are eager and enduring. The small knoll has many opportunities for variation, in height and width, which invite challenges suitable for each child’s resources. The children have visual, verbal and physical contact with each other. The top of the knoll provides an overview. Some find it scary the first time they try, but together they challenge each other, supporting and encouraging each other. The children decide how much they will participate and how they jump, and how they wish to solve the challenges offered by the knoll*” (Transcriptions). The physical activity was assessed at levels 5.

##### Interpretation

The physical character and the context (7Cs) of the knoll invite the children to play physically together. The physical and visual connections of the knoll (connectivity, 7Cs) promote physical activity but also afford social relations between the players. The knoll has variation in terms of height and flexibility (7Cs) which challenges the children to jump according to their individual abilities (where to jump from and where to land). The character, connectivity, change, clarity and challenges (7Cs) of the knoll perceive opportunities for different play affordances, invite social relations and promote high physical activity level. The characteristics of the knoll (not dangerous but challenging) allow different and open movement solutions, and involve the children according to their physical resources. This created moments of mastering and high physical activity. *The knoll plays with the children and the children play with the knoll, there is a mutual dynamic that generates movement and social togetherness.* The space of the knoll seems to challenges the physical and social resources of children to different play and lead to high physical activity. The staff should be familiar with the individual resources of the children, and should be willing to look for “physical playrooms” in nature which allow for social interaction.

In the situation with jumping from the knoll the preschool teacher demonstrated presence and entered into a physical community with the children:

At the moment the preschool teacher sees the activity is dying out, she instantly intervenes and leads the children to a more challenging section of the knoll. She grasps the correct moment, supporting, receiving and giving mutual responses through how the children express themselves in the activity. The play develops and the children experience moments of mastering (Transcriptions).

*Interpretation* The preschool teacher presence and skills in choosing the right instant to afford support and respond are important for the children’s activity levels, stamina and mastering in physical play. She made the children reach further than they would have otherwise done (scaffolding, Vygotsky). The staff’s ability to be present and willing may depend on such framework factors as the number of staffs, time, knowledge, attitudes, personal interest and the culture relating to physical play.

#### A day in the kindergarten’s outdoor play space

The findings show that in the outdoor play space high physical activity levels are created in particular sections/spaces of the outdoor area: on a small hill, and on the top of the hill by the playground equipment. This is where physical playing together involving several children arises (catch/tag, tobogganing/rolling down the hill). The area with the hill created different types of physical play that varied according to changes (7Cs) in the season, grass in summer and snow or ice in the winter. The playground at the top of the hill (slide, climbing castle, swings) offered views, overviews and large surfaces for moving. Two situations will be described below.

At the back of the day-care centre there is an area with a slope, stairs/fence and sandboxes. Seven children are standing, sitting and hanging on the fence on the slope. There are no adults involved with the children and no common organised activity.

Situation 4, free play (30 min); “*The children are involved with each other and in digging in the ground, playing with spades and buckets. Low staff involvement, little challenges, few common experiences. They are involved in activities for brief moments. No form of physically active play, the children are sitting, standing or walking to fetch or do things. No space is created for physical activity*” (Transcriptions). Physical activity is on level 2.

After an hour spent outdoors, they are allowed to go to the hill, by the playground equipment. Ten children aged three to five are there and one adult. A game of catch/tag is initiated. For periods the children are on a high level of physical activity based on their capabilities.

Situation 5, catch/tag game by the playground equipment (10 min); “The children are shouting ‘*X … can’t you catch us? Please catch us, try to catch us …*’. The staffs join the situation and run after the children. The children are shouting ‘*Catch me … can’t catch me*’ *… There is excitement and the staff are running after the children, catching them and holding them before releasing them. The staffs have high energy, the children focus on the adults, avoiding being caught. The adults show empathy, holding and hugging the child when it is caught. The game is exciting and creates enthusiasm. A high level of physical activity is created, by climbing up, sliding down, running around and hiding in the tower to escape capture by the adults. They run at high speed and the children’s body language shows that they are very much engaged in the game*” (Transcriptions). The physical level is assessed at level 4.5.

##### Interpretation

Situation 4, free play created a lower level of physical activity (level 2), while the situations 5, playing together, shared game initiated created a higher level of physical activity (level 4 or 5). The 3- to 5-year-olds create and initiate movement, but this indicates that the children do not themselves create a medium to high level of physical activity over an extended period of time; they need social affordances in character of invitations, imitations, responses, scaffolding and joint attention. Typical characteristics (7Cs) of the physical spaces in the kindergarten that generated physical play were overview, large spaces for moving, challenges, possibility of various types of playing, open opportunities for moving and space for several children (and adults) together to have physical-social contact.

## Discussion

Both the characteristics of physical and social affordances are factors influencing the level of physical activity. The findings reveal differences in the physical activity levels of children in a natural environment (level 4.4), compared to outdoor play space in the kindergarten (level 3.1). One explanation is that walking to and returning from trip destinations in a natural environment are physically demanding for many children (levels 3 or 4). Taking walks may encourage children who are otherwise not very physically active to have their day-to-day needs for physical activity satisfied. Another explanation is the character and context (7Cs) in a natural environment may offer other physical and social affordances, challenging the children in different ways than in the kindergarten’s context. According Fjørtoft (2000) the natural environments represent spaces where children see opportunities and challenges according to the qualities of the natural setting, and then use them functionally. The character and context (7Cs) of the nature environments can offer other types of sensory stimuli, less noise, different light and surfaces than what the kindergarten’s outdoor environment (Herrington and Brussoni 2015). Physical and social affordances may be changed and exchanged according to the unpredictable character of nature, more than in the familiar and habitual character of the kindergarten. The character (7Cs) of the natural environment create flexibility and open movement solutions, while fixed playground equipment, on the other hand, may call for closed or fixed ways of moving, which may be perceived as more predictable and boring. Chance and flexibility (7Cs) in natural elements is something that creates randomness and spontaneous exploration, manipulation and discovery, inviting all children into various types of playing based on the size, age and resources of the children (Herrington et al. 2007).

Children prefer environments that challenge their abilities and capacities while they are playing (Herrington and Brussoni 2015; Kyttä 2004; Spencer and Wright 2014). Sandseter (2009) refers to how the natural environment offers more challenges in children’s risky playing than in the kindergarten’s outdoor environment. Challenges (7Cs) and risk-taking are important for children’s health and development (Herrington and Brussoni 2015), and may help teach children about their own potential, and how they can navigate in new surroundings and master risk in different settings (Brussoni et al. 2015; Sandseter 2010). The challenges (7Cs) in the nature environment may challenge the tolerance of the participants for physical exploration and for what the staff will permit of children’s independent mobility licence. Findings showed that the staff’s involvement, regulation and tolerance of children’s independent mobility licence, regulates the possibilities to actualize high level of physical activity (situation 1 compared to situation 2). The findings reveal that natural environment offers potential affordances in forms of variation, challenges, various type of play, sensory stimulation, and different areas to move which are a catalyst for achieving a high physical activity level, depending on staffs tolerance of children’s mobility licence.

The findings indicated that physical environments affording social interaction are important for children’s duration and intensity in physical activity (situations 2, 3 and 5). Social affordances as invitations from others, responses, imitations, scaffolding and sharing moment of jointly fun are of significance of involvement and duration level of physical activity. Social affordances are opportunities of environments that catalyst interactions and social relationships in physical active play situations. Clark and Uzzell (2006) stated that individuals have to perceive its social meaning as important for the interactions. The jumping from a knoll situation in the nature and the catch/tag games showed that a change between the physical affordance (the terrain) and the social affordance (the staff and other children) challenged the children’s stamina and intensity in the play. Rietveld et al. (2013) maintain that we selectively respond to one affordance in favour of another. Social and physical affordances in environments can function as a zone of proximal development (Vygotsky), children are presented with a series of graduated zones of challenges, that promotes actions (Clark and Uzzell 2006). In the jumping from a knoll situation the children had the opportunity to manipulate the play themselves by deciding where they would jump from the knoll, how they jumped and who they jumped with. According to Gibson (1979) children see possibilities in what the environment offers, and interpret and use the environment they encounter in a functional manner based on their aptitudes and background. The knoll offered variation in height and width (three children jumped at the same time), and invited them into different types of play, social interaction, verbal, visual and physical contact. At and around the knoll a “physical social playroom” was created. The topography of the knoll affords social connectivity, interaction with friends across gender and age. The knoll challenges the social and physical resources of the children, and provides high stamina and physical activity levels. Imitation and joint attention may explain human involvement in an activity (Tomasello et al. 2005; Tomasello and Haberl 2003). Sharing social knowledge, observed behaviour, bodily attitudes and ways of being may motivate, challenge and “gear up” the body movements of the participants and thus their physical activity level. Processes of joint attention, broadly construed, play a critical role in the child’s socialization (Heft 2007). The staff should therefore be particularly attentive to children who are repeatedly excluded from physical play. Inviting all children to get involved in physical play together is important for the versatile development of children.

The observed kindergarten’s outdoor environment may be described as typically “ordinary”, reflecting many Norwegian kindergartens. It has an outdoor area of 3681.3 m^2^, varied terrain, access to playing equipment and natural material, thus it has *potentialaffordances* (Kyttä 2004). The findings show, however, that a physically inviting outdoor area is not necessarily adequate for 3- to 5-year-olds *themselves* to create a moderate to high level (level 4 or 5) of physical activity over time. Some 3- to 5-year-olds need to be social invited into physical jointly play, to *realisethe potential* affordances for physical activity. Bearing the recommendations from the Directorate (2014) in mind, suggesting an hour of a moderate to a high level of physical activity, it is perhaps disconcerting that most of the time the children spent outdoors in this study consisted of free play. There is no reason to believe that children personally create high levels of physical activity that satisfy the recommendations made by the Directorate of Health. In free play the physical activity level was relatively low (levels 1–3), with large individual variations. This indicates that physical activity is not always created by the children, but that it requires social invitations from others in the environment where they are playing. Playing together increased the physical activity level (levels 4 and 5), and more children were involved over a longer period of time compared to free play (levels 1–3). These findings encourage a discussion about the children’s free play in the kindergarten’s outdoor environment and about the extent to which this should be *challenged* and *balanced* by jointly play, initiated by adults to promote the physical activity levels of 3- to 5-year- olds. Educational content that promotes children’s level of physical activity is a challenge the staff should address. Kallestad and Ødegaard (2014) are concern of low level of planned activities (20 %) in relation to the preschool teacher’s professional role and the quality of the children’s learning. Physical games played together in inviting physical and social environments are attractive for most 3- to 5-year-olds.

## Educational implications in the kindergarten’s outdoor environment

The findings indicate that in physical play where the staff showed interest, expressed warmth and involvement in the physical world of the children, the children’s own involvement in and stamina for physical activity increased. The ability of the staff to enter into social interaction is important for the children’s development (Rogoff 2003; Vygotsky 1978). Children’s engagement and involvement in physical play changes from environmental affordances to the next so it is rarely possible to plan and have tight adult control on the activity. Being part of types of interaction through joint attention is an important part of being in the practice field (Tomasello et al. 2005). The staff should not direct or instruct a child on how to carry out the activity, but they should be guided in a sensitive manner, where the relationship between the adult and the child is mutual. The staff’s reflection upon their own tolerance for the children’s mobility licence is important aspects to focus on because their level of tolerance could impede or promote children’s physical play. Actualization of affordances involves a process of adjustment between children and their environment. Children’s involvement and participation in decision and planning of what and with who they want to actualizes physically active play is crucial.

## The study’s credibility

Several explanations for these findings may be posited. Random circumstances relating to the selected days, weather conditions, group size, content, staff’s education level, and the engagement of children and adults may have influenced the findings. The observations were only made in one kindergarten, so that generalising of the findings of children’s level of physical activity is challenging. At the same time the qualitative descriptions of characters of affordances in outdoors environments in relations to understand children’s level of physical activity enhancing an understanding of this topic. Qualitative observations highly depends on the observers who need to be sensitively, open and transparent. Using assistants was of great help for the researcher’s self-awareness in identifying the children’s levels of physical activity. The researcher considers the observations become more standardised by using the manual OSRAC-P and the criteria from 7Cs.

## Conclusions

The findings indicated a higher physical activity level in situations with physical play in a natural environment compared to kindergarten’s outdoor spaces, and in physical playing together situations. One conclusion is that the nature environments offer another character, context and atmosphere than kindergartens outdoors play spaces. The findings reveal that properties of the outdoors environment offering variation, challenges, opportunity for various types of play, sensory stimulation, and overview, large areas to move in, flexible materials, physical social connection opportunities and open movement solutions are positive for the physical activity level of 3- to 5-year-olds. The findings show, however, that a physically inviting outdoor area, thus it has potential affordances is not necessarily adequate for 3- to 5-year-olds themselves to create a moderate to high level of physical activity over time, that satisfy the recommendations made by the Directorate of Health. In free play the physical activity level was relatively low. Some 3- to 5-year-olds need to be social invited into physical jointly play, to realise the potential affordances for physical activity. The provision of environment that afford social interactions is important to reflect human interaction needs. Findings reveal that social relations, invitations into play, support, responses, scaffolding and social challenges offered by others, are probably catalysing duration and high level of physical activity.

The findings in this study supporting the 7Cs criteria and diverse affordances for play as qualities in outdoors play spaces (Herrington and Brussoni 2015). Children’s activity levels must be understood multi-dimensionally, as they are created in dynamic interaction between characteristics of the person-environment relationship. Future studies should examine in more detail properties of links between environmental architecture and social affordances.
